# A Comparison of Causative Pathogens in Bone and Prosthetic Joint Infections: Implications for Antimicrobial Therapy †

**DOI:** 10.3390/antibiotics13121125

**Published:** 2024-11-23

**Authors:** Annalise Unsworth, Bernadette Young, Matthew Scarborough, Martin McNally

**Affiliations:** 1 The Bone Infection Unit, Nuffield Orthopaedic Centre, Oxford University Hospitals, Oxford OX3 7HE, UK; annaliseunsworth@gmail.com (A.U.); bernadette.young@ouh.nhs.uk (B.Y.); matthew.scarborough@ouh.nhs.uk (M.S.); 2 St Vincent’s Hospital Clinical School, University of New South Wales, Sydney 2010, Australia

**Keywords:** fracture-related infection, FRI, bone infection, osteomyelitis, prosthetic joint infection, PJI, microbiology, antibiotic resistance

## Abstract

**Background**: The microbiological profile of bone and joint infections is important for determining the empiric choice of both systemic and local antimicrobial therapy. This study assessed whether there was a difference in the bacterial species that were isolated on culture in osteomyelitis (OM), fracture-related infection (FRI) or prosthetic joint infection (PJI). This was a retrospective, observational cohort study of patients who had surgical intervention for PJI or OM or FRI with a positive microbial culture between 2019 and 2022. **Methods**: Data including patient demographics, the site of injury, JS-BACH score, organism classification and antibiotic resistance to vancomycin and gentamicin were extracted from the medical records. **Results**: A total of 440 patients were included in this study: 163 patients with osteomyelitis, 109 with fracture-related infection with fixation implants and 168 with prosthetic joint infection. The patients with PJI were older, more likely to be female and had a higher BMI and ASA score compared to those with OM. Patients with PJI were more likely to have a higher JS-BACH score and more complex infections. *Staphylococcus aureus* was the most commonly isolated organism in all three groups. It was more frequently isolated in osteomyelitis than in PJI (*p* = 0.016). In both osteomyelitis and FRI, after *Staphylococcus aureus,* the next most common organisms were Gram-negatives, whilst for PJIs, the most commonly isolated organisms were *Staphylococcus aureus*, followed by coagulase-negative *Staphylococci* and then *Streptococcus* species. The rates of other organisms were broadly similar between the three groups. When adjusted for confounders, including symptom duration, JS-BACH score, the location of injury, age and BMI, there was no statistically significant difference in the presence of *Staphylococcus aureus* (OR = 0.765; 95% CI 0.633–1.232; *p* = 0.465) or polymicrobial infection (OR = 1.175; 95% CI 0.803–1.721; *p* = 0.407). **Conclusions**: Causative pathogens are similar across bone and joint infections and are independent of the presence of prosthetic material.

## 1. Introduction

Bone and joint infections cause significant morbidity to patients and pose high economic costs to healthcare systems [1,2,3]. The incidence of fracture-related infection is around 1% for closed fractures but up to 30% for open fractures [4], whilst the risk of prosthetic joint infection is 1–2% after primary procedures [5].

The management of bone and joint infections typically involves a combination of surgical resection and antibiotic treatment, delivered systemically and often with adjunctive local therapy [6,7,8]. The choice of empiric systemic and local antibiotic therapy is influenced by the underlying causative organisms and local antibiogram (the regional susceptibility of specific microorganisms to different antimicrobials [9]), and empiric treatment is typically continued until culture results are available [10].

The gold-standard microbiological test in the diagnosis of bone and joint infection is bacterial culture from deep tissue samples collected during surgical procedures [11,12]. *Staphylococcus aureus* is the most prevalent single pathogen in bone and joint infections due, in part, to biofilm formation and other pathogenic mechanisms in bone [13,14]. Culture-negative bone and joint infections occur in 1–16% [15,16] of PJIs and FRIs, which is thought to be due to previous antibiotic treatment, insufficient sample collection or difficult-to-grow organisms [15,17]. Therefore, an understanding of the most likely organisms is required to direct empiric treatment.

The choice of antimicrobial agent is important. In the past, patients were often treated for many weeks with broad-spectrum regimens [17,18,19,20]. However, there is increasing concern about antimicrobial resistance which, in 2021, was associated with an estimated 4.71 million deaths worldwide, and this is predicted to reach 8.22 million by 2050 [21]. Addressing this will require a better understanding of the microbiological profile in specific conditions to reduce unnecessarily broad antimicrobial use. Similarly, a reduction in adverse effects associated with antimicrobials is needed to improve patient compliance and experience [22,23,24].

Previous studies have described the individual microbiology of prosthetic joint infection, fracture-related infection and osteomyelitis [16,25,26] but have not compared all three. The aim of this study was to compare the microbiological epidemiology between the three groups at a tertiary referral hospital in the United Kingdom. We also investigated the influence of the implant material (joint prostheses or fracture fixation devices) on the microbiological profile.

## 2. Results

**Figure 1:**
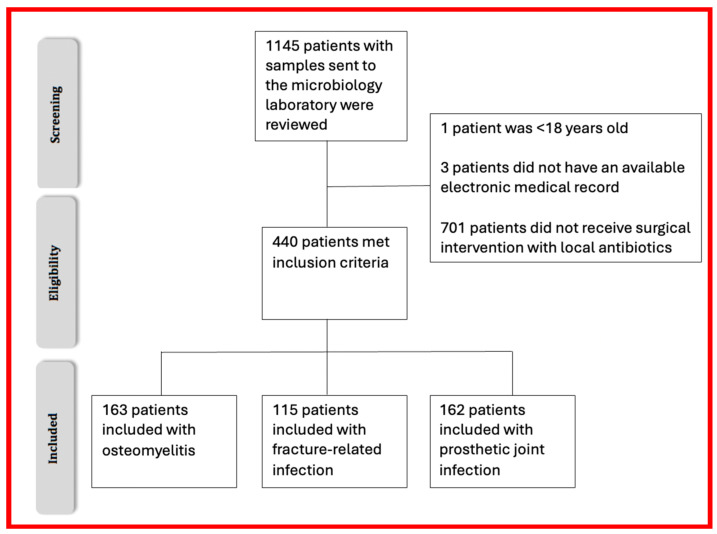
Patient inclusion flow diagram.

A total of 1145 patients with bone and joint infections were reviewed. Of these, 440 met the inclusion criteria and were included in the study analysis. There were 163 in the osteomyelitis-alone group, 115 with fracture-related infection and 162 in the prosthetic joint infection group. All had implantation of local antimicrobials in a licenced carrier, usually with gentamicin and/or vancomycin (Figure 1).

Patients with prosthetic joint infection were typically older, were more likely to be female, had a higher BMI and were more likely to have other comorbidities with a higher American Society of Anesthesiologists Score (ASA score) (Table 1). Hip and knee infection were the most common PJIs, whilst long bone infections (predominantly tibia and femur) were the most common osteomyelitis and FRIs. Generally, all groups presented most often as chronic infections in patients with significant comorbidities (JS-BACH complex) (Table 2).

Table 3 describes the differences in the microbiological characteristics of the three groups. *Staphylococcus aureus* was the most commonly isolated organism in all groups but was proportionately more common in osteomyelitis than in prosthetic joint infection (*p* = 0.014). In both osteomyelitis and fracture-related infection, after *Staphylococcus aureus*, the next most common organisms were Gram negatives, whilst for prosthetic joint infections, the most commonly isolated organisms after *Staphylococcus aureus* were coagulase-negative *Staphylococci* and then *Streptococcus* species.

There was a significant difference in the presence of polymicrobial infection between the three groups (*p* = 0.013). Polymicrobial infection was more likely in osteomyelitis (36.2%) compared to prosthetic joint infection (21.6%) (*p* = 0.012), but there was no difference in the polymicrobial infection rates between fracture-related infection and the other two groups (*p* = 0.171 between FRI and PJI and *p* = 1.0 between FRI and OM). Of the patients with fracture-related infection, 37/115 (32.2%) had polymicrobial infection. Monomicrobial infection was relatively more common in FRIs presenting early, within 4 weeks of fracture (14/21; 66.6%), or in chronic cases with a duration of infection of more than 12 weeks (59/83; 71.1%). Polymicrobial infection was most common in FRIs with a shorter duration (4–12 weeks) (6/11; 54.5%).

There was no difference in the presence of gentamicin- or vancomycin-resistant organisms between the three groups. The rate of laboratory confirmed gentamicin resistance was 8.6% in osteomyelitis, 11.3% in FRI and 9.9% in PJI (*p* = 0.754), and the rate of confirmed vancomycin resistance was 2.5% in OM, 2.6% in FRI and 0% in PJI (*p* = 0.126). There was no difference in combination gentamicin and vancomycin resistance between the three groups. When adjusted for confounders, including symptom duration, JS-BACH score, the location of injury, age, ASA and BMI, there was no difference in the presence of *Staphylococcus aureus* (OR = 0.765; 95% CI 0.633–1.232; *p* = 0.465), *Streptococcus* species (OR 0.561; 95% CI 0.313–1.007; *p* = 0.053) or polymicrobial infection (OR = 1.175; 95% CI 0.803–1.721; *p* = 0.407) (Table 4).

### Prosthetic Material Compared to No Prosthetic Material

Table 5 describes differences between prosthetic material compared to no prosthetic material in bone and joint infection.

*Staphylococcus aureus* was more likely in osteomyelitis than in infections with prosthetic material (62.6% versus 49.1%, *p* = 0.006). The presence of polymicrobial infection was 36.2% in osteomyelitis compared to 26% in patients with prosthetic material (*p* = 0.024). When adjusted for confounders, there was no difference in the presence of *Staphylococcus aureus* (OR = 0.654; 95% CI 0.402–1.066; *p* = 0.088) or polymicrobial infection (OR = 0.687; 95% CI 0.410–1.150; *p* = 0.153).

## 3. Discussion

This study compared the microbiology of osteomyelitis, FRI and PJI at a tertiary bone and joint infection unit. *Staphylococcus aureus* was the most commonly isolated organism in all three groups, in keeping with previous studies [13,14]. There were more cases of *Streptococcus* infection in PJI than in OM or FRI. These findings are similar to those of Rupp et al. [27], who compared a smaller series of FRIs and PJIs. They also found no statistical difference in the distribution of pathogens but slightly more difficult-to-treat organisms in PJI. The similarity of microbiological organisms across different types of bone infection supports the standardization of empiric antibiotic treatment regimes in bone and joint infection.

Empiric regimes have often been based on the assumption that bone and joint infections are predominantly Gram-positive, with limited need for Gram-negative cover. Also, regimes have been different in Trauma Units, which treat fractures and fracture-related infections, compared to elective Orthopaedic Units, where prosthetic joint replacement is performed, and osteomyelitis is treated. Multiple antimicrobial policies can be confusing for treating staff.

This study suggests that the microbiology is not significantly different, and that polymicrobial infection, including Gram-negative organisms, is a common occurrence. Simplifying empiric antimicrobial regimes based on these findings may improve antimicrobial stewardship and compliance in hospitals.

We previously studied empiric systemic antimicrobial regimes separately in osteomyelitis and fracture-related infection, using different patient cohorts [28,29]. This new study provides similar evidence to indicate that an initial empiric therapy should be broad spectrum, such as a glycopeptide and anti-pseudomonal Gram-negative cover. In our unit, we recommend a short period of vancomycin and meropenem intravenously, starting after microbiological sampling in the theatre. This is rationalized to more specific therapy when culture results are available, usually beginning at 48 h after surgery.

Data from previously published cohorts show a similar distribution of bacterial species, but with small differences, particularly when comparing the United Kingdom with continental Europe. Table 6 and Table 7 show the distribution of bacteria in studies on PJI and FRI from the UK, Germany, France and Belgium [17,26,27,30]. The UK cohorts are more similar in terms of FRI microbiology, with the exception of the incidence of *Staph. aureus,* which was still the most common pathogen. In PJI, the microbiology was very similar, including polymicrobial infections and gentamicin and vancomycin resistance.

### 3.1. Polymicrobial Infections

The presence of polymicrobial infection was consistently high across the three groups: 36% in osteomyelitis, 32% in FRI and 22% in PJI. Polymicrobial infections are more common in patients with tissue ischemia or in an immunocompromised host [14]. Additionally, in a large French registry study of osteomyelitis, the rate of polymicrobial infection was 30% [31], and the rate of polymicrobial infection of fracture-related infection patients in a UK trauma centre was 34% [26]. This is higher than the incidence reported by Rupp et al. [27], who found 10–17% of polymicrobial infections in FRI and PJI. The presence of polymicrobial infection in haematogenous osteomyelitis is possibly associated with the chronicity of infection. In our cohort, 97% of those with osteomyelitis had a duration of infection greater than 12 weeks. This may facilitate the introduction of secondary bacteria or skin colonizers via a sinus tract or due to superimposed infection of necrotic or devascularized bone [14].

Whilst fracture-related infection is not a new entity, the consensus definition for fracture-related infection is recent, and a knowledge gap regarding empirical treatment for FRI exists [17]. Depypere et al. described the microbiological aetiology of FRI in Belgium. Polymicrobial infection was present in 25% and was more likely in early FRI (<14 days) [17]. Similarly, Corrigan et al., in a multicentre study from the UK and the Netherlands, noted an increased likelihood of polymicrobial infection in early (<2 weeks) and delayed (3–10 weeks) FRI compared to late infection (>10 weeks) [10]. In our patients with FRI and prosthetic material, there was no difference in early (<4 weeks) compared to late (>12 weeks) FRI for the presence of polymicrobial infection. However, culture-negative FRIs were not included in our cohort, and the definitions of the duration of infection are different between the studies. The presence of polymicrobial infection at all time points suggests that assumptions regarding microbiological epidemiology should not be used to guide antimicrobial therapy [10].

### 3.2. Local Antibiotic Treatment Implications

Local therapy is now a common component of treatment for FRI, PJI and OM, usually with implantation of aminoglycoside with or without a glycopeptide in bioabsorbable carriers or in polymethylmethacrylate (PMMA) cement [6,9,31]. The rate of confirmed gentamicin resistance was between 9 and 12% in our study, with no difference between the three groups. The confirmed vancomycin resistance was 2.5–2.6%. In our cohort of patients, there was no difference in the presence of gentamicin or vancomycin resistance between patients with prosthetic material—either FRI or PJI—compared to those who had osteomyelitis without implants (Table 5). The confirmed resistance rate of combination gentamicin and vancomycin was low (zero for PJI, 1.8% for FRI and 2.5% for osteomyelitis). This is similar to the resistance rates for PJI and FRI presented by Rupp et al. [27]. However, the microbiological isolates were not tested for aminoglycoside or vancomycin susceptibility if there were no EUCAST breakpoints for these organisms [32]. Examining for presumed resistance, the combined gentamicin and vancomycin resistance rate was much higher, at 11%, although again, there was no difference between osteomyelitis, prosthetic joint infection or fracture-related infection.

Previous studies at our centre demonstrated no clinical difference in outcomes with local gentamicin alone compared to the combination of local gentamicin and vancomycin, and there was no difference in outcomes in patients with gentamicin resistance in osteomyelitis and FRI [33,34]. The presence of similar resistance patterns between the groups may support a unified local antibiotic treatment approach for both PJI and OM/FRI.

### 3.3. Limitations

The main limitation of this study was that it is a single-centre retrospective review. Thus, the microbiological epidemiology is representative only of the single centre and may not be able to be extrapolated to other regions. However, as a tertiary referral centre, the Bone Infection Unit receives patients referred from all over the United Kingdom and likely represents a wider epidemiological base than just the local catchment. Additionally, prior antibiotic treatment history was not consistently available to be included in this study and may have affected the culture results.

In this study, we did not collect details of the duration of infection prior to treatment. This may have affected the microbiological profile. However, in a previous study of 433 FRIs, we showed that the microbiology was not affected by the duration of infection [10]. This issue has not been studied in PJI and would be an interesting future study.

## 4. Materials and Methods

This was a retrospective observational cohort study at Oxford University Hospital Bone Infection Unit. The Bone Infection Unit is a specialized unit treating adult patients from the United Kingdom with musculoskeletal infections, including fracture-related infection, prosthetic joint infections and haematogenous infections of the bones, joints and spine.

### 4.1. Recruitment and Inclusion Criteria

The medical and surgical records and laboratory details of all patients who were admitted for treatment to the Bone Infection Unit between January 2019 and September 2022 were reviewed. We identified all consecutive patients with a diagnosis of prosthetic joint infection, osteomyelitis and fracture-related infection who were also treated surgically. Individual patients were only included once. Recurrent infections were not included. Patients who were under 18 years old at the time of treatment were excluded. Patients were only included if they had implantation of antibiotic carriers as part of their surgical management.

Infection was confirmed using the International FRI Consensus Definition [35] and the European Bone and Joint Infection Society (EBJIS)’s definition of Prosthetic Joint Infection [36]. FRI included only patients who had confirmatory criteria from the consensus definition and had fracture fixation implants in place at the time of treatment. Osteomyelitis patients had haematogenous osteomyelitis or had a previous history of injury (contiguous focus osteomyelitis) but did not have implants in place at the time of treatment. Patients were only eligible for inclusion if five or more separate tissue samples were taken at surgery for microbiological culture. If implants were removed at surgery, these could also be sent for culture of fluid after sonication [37], but this was not mandatory. The culture protocol has previously been reported [38]. Infection was confirmed with at least two positive cultures of a phenotypically identical organism. A single positive culture was accepted when there was also confirmatory evidence of infection on the histopathology. Tissue samples for histology (usually 3 or more) were embedded in paraffin and 5 µm sections were cut. The sections were stained with haematoxylin and eosin and Gram-stained. At least 10 high power fields were examined per section (×400). Positive histology was defined as the presence of visible microorganisms on the Gram stain or an average of ≥5 polymorphonuclear neutrophils seen per high power field on haematoxylin and eosin-stained sections [28,39,40].

### 4.2. Microbial Sampling

All patients had at least five samples sent intraoperatively for microscopy and culture. Antibiotics were stopped at least two weeks before surgery, provided that the patient was clinically stable, without evidence of bacteraemia. Surgery was performed under tourniquet where possible. In all cases, deep samples were taken by a validated protocol [28] involving the harvest of at least 5 specimens of tissue, each taken with clean instruments and avoiding contact with the skin. Samples were transferred immediately to the microbiology laboratory for processing. Samples were disrupted in 3 mL saline, using sterile glass beads, with vortexing for 15 min at 40 Hz. Then, 0.5 mL of the fluid sample was inoculated into a BD BACTEC™ Lytic/10 Anaerobic/F bottle (Franklin Lakes, NJ, USA), and a further 0.5 mls were placed into a BACTEC™ Plus Anaerobic/F bottle. Incubation was for 10 days, with sub-culture of flagged positive bottles. If no growth was flagged at 10 days, a terminal culture was performed onto fastidious anaerobic agar and lysed blood “chocolate” agar [41].

### 4.3. Data Collection

Medical records were reviewed for inclusion and exclusion criteria, and data were extracted using a standardized template. Demographic information collected included age, gender, Body Mass Index (BMI) and American Society of Anaesthesiology (ASA) score [42]. Clinical data collected included the location of infection, Joint-Specific, Bone Involvement, Antimicrobial options, Coverage of the soft tissues, Host status (JS-BACH) classification [43,44], symptom duration, microbiological characteristics and antimicrobial susceptibility profile.

We specifically collected data on the presence of resistance to the antibiotics which are most frequently used in local therapy (vancomycin and gentamicin). These are often used empirically, particularly in FRI, with no prior knowledge of the microbiological diagnosis. Laboratory-tested resistance to vancomycin or gentamicin was confirmed using MICs and European Committee on Antimicrobial Susceptibility Testing (EUCAST) breakpoints [32]. Presumed gentamicin and vancomycin resistance was based on the Sanford guide antibiogram. If the antibiotic was classified as not recommended by the Sanford guide for that particular microorganism, it was categorized as resistant [45].

### 4.4. Data Management and Analysis

Analysis was performed using IBM SPSS v29. The difference in the microbiological organism between the three groups (osteomyelitis, fracture-related infection and prosthetic joint infection) were compared using a one-way ANOVA. Post hoc testing used an independent samples t test, and Bonferroni correction was used to determine the difference. Potential confounders considered for inclusion in the regression models were age, BMI, the location of injury, JS-BACH score and symptom duration. Each potential confounder was examined separately by univariate analysis to determine their association with the outcome variable. Confounders whose association with the outcome variable had a *p* value < 0.2 were included in the multivariate model.

## 5. Conclusions

Causative pathogens are similar across bone and joint infections and are independent of the presence of prosthetic material. There was no significant difference in the identification, presence of polymicrobial infection or gentamicin and vancomycin resistance in organisms that were isolated in osteomyelitis or fracture-related infection compared to prosthetic joint infection. This may have implications for the development and standardization of empiric antibiotic regimens and local antibiotic therapy in the management of bone and joint infections.

## Figures and Tables

**Table 1:** Baseline patient demographics.

Characteristic	Osteomyelitis (n = 163)		Fracture-Related Infection (n = 115)		Prosthetic Joint Infection (n = 162)	
	Median	IQR *	Median	IQR *	Median	IQR *
Age (years)	56	37–66	56	41–64	70	60–78
ASA *	2	1–3	2	1–3	3	2–3
BMI *	26	22–31	27	24–31	30	25–36
	***n***	**%**	***n***	**%**	***n***	**%**
Sex (Male)	125	76.7	75	65.2	72	44.4
**Site of Infection**						
Long Bone	135	82.8	91	79.1	0	0
Hand and wrist	5	3.1	1	0.9	1	0.6
Foot and ankle	16	9.8	10	8.7	2	1.2
Hip	2	1.2	0	0	79	48.8
Knee	1	0.6	1	0.9	75	46.3
Spine	3	1.8	12	10.4	0	0
Elbow	0	0	0	0	3	1.9
Shoulder	1	0.6	0	0	2	1.2

* IQR: Inter-quartile range; ASA: American Society of Anaesthesiology; BMI: Body Mass Index.

**Table 2:** Infection characteristics.

Characteristic	Osteomyelitis (n = 163)		Fracture-Related Infection (n = 115)		Prosthetic Joint Infection (n = 162)	
	***n***	***%***	***n***	***%***	***n***	***%***
JS-BACH *						
Uncomplicated	57	35.0	40	34.8	27	16.7
Complex	104	63.8	72	62.6	124	76.5
Limited Options	2	1.2	3	2.6	11	6.8
**Duration of Infection**						
<4 weeks	3	1.8	21	18.3	45	27.8
4–12 weeks	2	1.2	11	9.6	10	6.2
>12 weeks	158	96.9	83	72.2	107	66

* JS-BACH: Joint-Specific, Bone Involvement, Anti-microbial options, Coverage of the soft tissues, Host status classification.

**Table 3:** Microbiological characteristics.

Characteristic	Osteomyelitis (*n* (%))	Fracture-Related Infection (*n* (%))	Prosthetic Joint Infection (*n*, (%))	*p* Value
**Organism classification**				
*Staphylococcus aureus*	102 (62.6)	60 (52.2)	76 (46.9)	0.016
Coagulase-negative *staphylococcus*	26 (16.0)	23 (20.0)	34 (21.0)	0.479
*Streptococcus* species	19 (11.7)	9 (7.8)	28 (17.3)	0.058
*Enterococcus* species	10 (6.1)	11 (9.6)	14 (8.6)	0.046
*Pseudomonas* species	13 (8.0)	8 (7)	8 (4.9)	0.537
Other Gram negatives	37 (22.7)	27 (23.5)	27 (16.7)	0.282
Other Gram positives	28 (17.2)	15 (13.0)	13 (8.0)	0.537
*Candida* species	0	1 (0.9)	1 (0.6)	0.530
Gram-positive organisms only	118 (72.4)	77 (67.0)	126 (77.8)	0.134
Gram-negative organisms only	12 (7.4)	9 (7.8)	16 (9.9)	0.694
Polymicrobial infection	59 (36.2)	37 (32.2)	35 (21.6)	0.013

**Table 4:** Resistance profiles.

	Osteomyelitis (*n* (%), 95% CI)	Fracture-Related Infection (*n* (%), 95% CI)	Prosthetic Joint Infection (*n*, (%), 95% CI)	*p* Value
**Resistance**				
Confirmed gentamicin resistance	14 (8.6), 4.3–13.5	13 (11.3), 6.1–17.4	16 (9.9), 5.6–14.8	0.754
Presumed gentamicin resistance	2 (17.8), 12.3–24.5	25 (21.7), 13.9–29.6	20 (12.3), 7.4–17.3	0.110
Confirmed vancomycin resistance	4 (2.5), 0.6–4.9	3 (2.6), 0–6.1	0	0.126
Presumed vancomycin resistance	44 (27.0), 20.2–33.7	34 (29.6), 21.7–38.3	31 (19.1), 13–25.9	0.100
Confirmed gentamicin and vancomycin resistance	4 (2.5), 0.6–4.9	2 (1.7), 0–4.3	0	0.150
Presumed gentamicin and vancomycin resistance	18 (11.0), 6.1–16.6	13 (11.3), 6.1–17.4	17 (10.5), 6.2–15.4	0.975

**Table 5:** Prosthetic material (PJI + FRI) compared to bone infection without implanted metalware (osteomyelitis).

Characteristic	Osteomyelitis (*n* (%))	Metalwork In Situ (PJI + FRI with Metalwork)	*p* Value
**Organism Classification**			
*Staphylococcus aureus*	102 (62.6)	136 (49.1)	0.006
Coagulase-negative *staphylococcus*	26 (16.0)	57 (20.6)	0.231
*Streptococcus* species	19 (11.7)	37 (13.4)	0.605
*Enterococcus* species	10 (6.1)	25 (9.0)	0.279
*Pseudomonas* species	13 (8.0)	16 (5.8)	0.369
Other Gram negatives	37 (22.7)	54 (19.5)	0.423
Other Gram positives	28 (17.2)	28 (10.1)	0.537
*Candida* species	0	2 (0.7)	0.396 *
Polymicrobial infection	59 (36.2)	72 (26.0)	0.024
Confirmed vancomycin + Gentamicin resistance	4 (2.5)	2 (0.7)	0.139 *

* Fisher’s exact test used as less than 5 in each cell; PJI: prosthetic joint infection; FRI: fracture-related infection.

**Table 6:** The percentage of each pathogen in prosthetic joint infection.

Comparison Site	This Study *n* = 162 2024, UK	Rupp et al. [27] *n* = 81 2021, Germany	Triffaut-Fillit et al. [30] *n* = 567 2019, France
*Staphylococcus aureus*	46.9	27.9	28.9
Coagulase-negative *Staphylococci*	21.0	23.3	28.6
*Streptococcus* spp.	17.3	10.5	13.1
Gram negatives	21.6	10.5	Not reported
Polymicrobial infection	21.6	17.3	18.2
Combined gentamicin and vancomycin resistance	10.5	9.9	Not reported

**Table 7:** The percentage of each pathogen in fracture-related infection.

Comparison Site	This Study *n* = 115 2024, UK	Patel et al. [26] *n* = 294 2023, UK	Rupp et al. [27] *n* = 86 2021, Germany	Depypere et al. [17] *n* = 191 2022, Belgium
*Staphylococcus aureus*	52.2	24.4	37.4	31.4
Coagulase-negative *Staphylococci*	20	14.0	16.9	25.8
*Streptococcus* spp.	7.8	4.5	7.2	‘rarely detected’
Gram negatives	30.4	39.7	10.5	27.8
Polymicrobial infection	32.2	34.2	10.5	25.3
Combined gentamicin and vancomycin resistance	11.3	5.8	6.8	Not reported

## Data Availability

The data presented in this study are available on request from the corresponding author due to privacy reasons.
